# Up-regulation of heat shock protein 27 inhibits apoptosis in lumbosacral nerve root avulsion-induced neurons

**DOI:** 10.1038/s41598-019-48003-9

**Published:** 2019-08-07

**Authors:** Zhi-bin Zhou, Gao-xiang Huang, Jia-jia Lu, Jun Ma, Qi-jun Yuan, Yan Cao, Lei Zhu

**Affiliations:** 1 Department of Orthopedic Trauma Surgery, Changzheng Hospital, Second Military Medical University, Shanghai, 200003 China; 2 Department of Pathology, The 924th Hospital of the Chinese People’s Liberation Army Joint Logistic Support Force, Guangxi Key Laboratory of Metabolic Diseases Research, Guilin, Guangxi 541002 China; 3 Department of Orthopedics, The 85th Hospital of the Chinese People’s Liberation Army, Shanghai, 200052 China

**Keywords:** Molecular medicine, Neurodegeneration

## Abstract

Lumbosacral nerve root avulsion leads to widespread death of neurons in the anterior horn area of the injured spinal cord, which results in dysfunction in the lower extremities. Heat shock protein 27 (Hsp27) has been found to play cytoprotective roles under adverse conditions. However, the role of Hsp27 in neurons after lumbosacral nerve root avulsion is unknown. The aim of the present study was to investigate the effects and mechanism of action of Hsp27 on neurons after lumbosacral nerve root avulsion. It was found that Hsp27 expression was elevated in the anterior horn area of the injured spinal cord and the up-regulation of Hsp27 protected neurons against apoptosis after lumbosacral nerve root avulsion. In addition, Hsp27 plays an anti-apoptotic role by suppressing oxidative stress reactions. These findings indicated that Hsp27 may play a key role in resistance to lumbosacral nerve root avulsion-induced neuron apoptosis and may prove to be a potential strategy for improving prognosis after lumbosacral nerve root avulsion.

## Introduction

Lumbosacral nerve root avulsion causes the physical disconnection of nerve roots from the spinal cord, which leads to widespread neuron death in the anterior horn area of the injured spinal cord^1^. This severe neuron death results in devastating motor dysfunction, neuropathic pain, and numbness in the lower extremities^2–5^. In recent years, surgeons have developed novel surgical concepts to treat injuries related to lumbosacral nerve root avulsion such as immediate reimplantation and contralateral nerve root transfer in patients and experimental animals, but functional recovery remains poor^5–8^. One of the most important factors leading to the poor prognosis is the death of neurons after lumbosacral nerve root avulsion. Our previous study demonstrated that apoptosis occurred in the neurons in the corresponding anterior horn of the spinal cord after lumbosacral nerve root avulsion, contributing to the decreased neuron survival rate^9^. Methods to inhibit neuron apoptosis may improve the prognosis of lumbosacral nerve root avulsion injuries.

Heat shock protein (Hsp) family represents a group of stress-responsive proteins in various cell types^10–12^. Papadopoulos^13^ *et al*. showed that selective expression of Hsp70 enhanced the survival of astrocytes under oxygen-glucose deprivation. Huang^14^ *et al*. showed that hyperbaric oxygen preconditioning protected rat spinal neurons against oxidative injury and oxygen-glucose deprivation by up-regulating of Hsp32. In addition, Hsp27 can protect cells against apoptotic death in response to cellular stressors including ischemia, heat shock, and oxidative stress, and has been shown to interact with important apoptotic proteins and to block apoptotic pathways^15^. He^16^ *et al*. found that only Hsp27-positive large motor neurons survived at one week after ventral root avulsion. Hsp27-negative small motor neurons were more vulnerable to avulsion than Hsp27-positive large motor neurons, suggesting a protective role of Hsp27 after avulsion. However, the exact role of Hsp27 in neurons and apoptosis after lumbosacral nerve root avulsion remains unclear.

The aim of our study was to reveal whether the expression of Hsp27 was upregulated after lumbosacral nerve root avulsion and oxygen-glucose deprivation (OGD). Moreover, the study aimed to show the anti-apoptotic effects and mechanism of action of Hsp27 under these conditions. The findings suggested that Hsp27 inhibited apoptosis and may prove to be a strategy for improving prognosis after lumbosacral nerve root avulsion.

## Materials and Methods

### Animals and establishment of the lumbosacral nerve root avulsion model

All animal experiments strictly followed the guidelines of the Animal Ethics Committee of the Second Military Medical University (Shanghai, China). All animal experiment protocols also were approved by the Animal Ethics Committee of the Second Military Medical University (Shanghai, China). Adult male Sprague-Dawley (SD) rats (200–220 g) were purchased from the Shanghai Laboratory Animal Center (SLAC) and were housed under a 12-hour light/dark cycle in a specific pathogen-free environment with standard temperature and free access to food and water. Twenty rats were randomly divided into 5 groups (N = 4 in each group). The lumbosacral nerve root avulsion surgery was performed in rats as described previously^17^, and the avulsed rats were sacrificed at 1 d, 3 d, 5 d or 14 d after surgery. Briefly, after anesthesia (1% pentobarbital, 40 mg/kg, intraperitoneal injection) and routine disinfection, the right L4–L6 nerve roots were exposed and avulsed with a tiny self-made hook under an operation microscope of 10× magnification. In the sham group, the right L4–L6 nerve roots of rats were only exposed but not avulsed. The incision was sutured and disinfected regularly after the operation.

### Adenoviral infection and animal groups

The recombinant adenovirus carrying short hairpin RNA (shRNA) targeting rat Hsp27 (Ad-shHsp27) or Hsp27 expression plasmid (Ad-Hsp27), and the control adenovirus containing a scrambled shRNA (Ad-scramble) or empty plasmid (Ad-vector) were constructed and purified by Hanbio Biotechnology Co., Ltd (Shanghai, China). Three shRNAs targeting Hsp27 and scramble shRNA were designed by Shanghai GenePharma Co., Ltd (China). The knock-down effects of these shRNAs were validated (data not shown) and the sequences of the most efficient shRNA were as follows: Hsp27 forward primer, caccGCTGGGAAGTCTGAACAGTTTCAAGAGAACTGTTCAGACTTCCCAGC; Hsp27 reverse primer, aaaaGCTGGGAAGTCTGAACAGTTCTCT. The scramble control shRNA sequence was TGAAACTGTTCAGACTTCCCAGC. Rats (N = 16) were randomly divided into 4 groups (Sham combined with Ad-scramble group, Sham combined with Ad-shHsp27 group, spinal root avulsion combined with Ad-scramble group, spinal root avulsion combined with Ad-shHsp27 group, N = 4 in each group). Laminectomy was performed at the L4–L6 level, leaving the dura intact and six μl of solution containing 6 × 10^9^ infective units of adenovirus (Ad-scramble or Ad-shHsp27) was injected into the right stumps of the L4–L6 spinal cord with a Hamilton microsyringe. Two μl of adenovirus was injected into each stump and a total of 6 μl of adenovirus was injected into each rat. Then, the right L4–L6 nerve roots were avulsed in the avulsion group. The right L4–L6 spinal cord was resected for further assay 3 days after surgery.

### Cell line culture

Human neuroblastoma SH-SY5Y cells (ATCC, CRL-2266) were cultured in DMEM/Ham’s F-12 with glutamine (Gibco, Grand Island, NY, USA), supplemented with 10% fetal bovine serum and 1% penicillin-streptomycin. The cell culture was maintained at 37 °C and 5% CO_2_ in an incubator. The medium was replaced twice a week. The cells were used to perform further assays at passage number 30.

### Primary neuron culture

Primary neuron was isolated and cultured as our previous study^17^. Briefly, the spinal cord from embryonic day 14–15 rats was cut into 1 mm^3^ pieces. The pieces were then digested in 0.05% trypsin for 20 min at 37 °C. The digestion was ceased with 2 ml DMEM (Invitrogen, Carlsbad, CA, USA) supplemented with 10% heat-inactivated fetal bovine serum (FBS) and 5% heat-inactivated horse serum (Invitrogen, Carlsbad, CA, USA) at room temperature. After centrifugation, supernatant was removed. The tissue was re-suspended and re-digested according to the procedures described above. The supernatant containing single cells was collected and diluted to the indicated density. The cells were plated into a poly-L-lysine-coated (PLL, Sigma, St. Louis, MO, USA) plate. Four hours later, the medium was replaced with serum-free neurobasal medium (Invitrogen, Carlsbad, CA, USA) supplemented with 2% B27 supplement (Invitrogen, Carlsbad, CA, USA) and 2 mM glutamine (Invitrogen, Carlsbad, CA, USA). At next day, 5 μM cytosine-β-D-arabinofuranoside (Sigma, St. Louis, MO, USA) was added into the medium to inhibit non-neuronal cell division. Fresh medium was replaced twice a week. After a week, primary neurons were used for the indicated experiment.

### Oxygen-glucose deprivation (OGD) treatment

For OGD treatment, primary neurons and SH-SY5Y cells were plated at a density of 6 × 10^5^ cells/well on 6-well plates. When the experiment began, the culture medium was replaced with serum-free and glucose-free DMEM and then the cells were placed in a hypoxic incubator chamber (1% O_2_, 5% CO_2_, 94% N_2_; Forma Scientific, USA) for the indicated hours. Control cells were cultured with complete medium and were incubated under normal oxygen tension (20% O_2_, 5% CO_2_).

### Western blotting analysis

Rats were terminally anesthetized at 1, 3, 5, and 14 days after avulsion. Whole protein extracts from the injured spinal cord, primary neurons and SH-SY5Y cells were prepared as described previously^17^. After electrophoresis, proteins were transferred to a nitrocellulose membrane, blocked with 5% nonfat milk, and probed overnight with primary antibodies against goat Hsp27 antibody (Santa Cruz Biotechnology, Santa Cruz, CA), rabbit p-p65 antibody (Ser536) and mouse GAPDH antibody (Cell Signaling, Boston, MA, USA). The membranes were washed three times and incubated with horseradish peroxidase-conjugated secondary antibodies (1:5000, Rockland Immunochemicals, Limerick, PA, USA) for 2 h. Finally, blots were detected by ECL chemiluminescence (Pierce, Rockford, IL, USA). Protein bands were quantitated with ImageJ software (NIH, USA) using GAPDH as an internal control.

### Immunofluorescent staining

For tissue preparation, the rats were perfused transcardially with 4% paraformaldehyde (PFA). The L4–L6 spinal cord, including the avulsion epicenter, was resected and fixed with 4% PFA overnight and embedded in paraffin. Consecutive serial sections (4 μm) were deparaffinized, rehydrated, blocked in PBS with 3% bovine serum albumin (BSA) and incubated with rabbit anti-Hsp27 antibodies (#2442; Cell Signaling Technology, USA) and rabbit anti-NeuN (#54761; Cell Signaling Technology, USA), diluted in PBS overnight at 4 °C. After rinsing with PBS, the sections were incubated with goat anti-rabbit IgG Alexa Fluor 594 and 488 secondary antibodies (Molecular Probes, Eugene, Oregon, USA) for 2 h at room temperature, and mounted with Vectashield containing DAPI to label the nuclei. For cell staining, SH-SY5Y cells were fixed with 4% PFA for 30 min following the indicated treatment, washed three times in 0.01 M PBS, and blocked with 10% BSA for 20 min at room temperature. The following protocols were the same as in the tissue staining section.

### Transient cell transfection

For cell line transfection, SH-SY5Y cells were seeded on 6-well plates at a density of 6 × 10^5^ cells/plate and cultured overnight. The SH-SY5Y cells were transfected with Hsp27 shRNA plasmid (sc-29350-SH, Santa Cruz Biotechnology) or control shRNA plasmid (sc-108060, Santa Cruz Biotechnology) using Lipofectamine 2000 Transfection Reagent (Invitrogen) according to the manufacturer’s protocol when at ~80% confluence for 72 hour. For adenoviral transient infection, primary neurons were infected with the recombinant adenovirus carrying scramble RNA (Ad-scramble), short hairpin RNA-Hsp27 (Ad-shHsp27), empty vector (Ad-vector) or Hsp27 expression plasmid (Ad-Hsp27) at a multiplicity of infection of 100 for the indicated hours. The knockdown efficiency was determined by immunofluorescence staining or western botting.

### Terminal deoxynucleotidyl transferase dUTP nick end labeling (TUNEL) staining

Paraffin-embedded tissues were sectioned consecutively (4 μm), and the sections were deparaffinized, rehydrated. Primary neurons were fixed with 4% PFA for 30 min following the indicated treatment, washed three times in 0.01 M PBS. Then tissue sections and neurons were blocked in PBS with 3% bovine serum albumin (BSA), and stained using the ApoBrdU DNA Fragmentation Assay Kit (BioVision, Mountain View, CA, USA) according to the manufacturer’s protocol.

### Apoptosis detection by flow cytometry

Following transient transfection for the indicated hours, SH-SY5Y cells and neurons were treated by OGD for different hours. SH-SY5Y cells and neurons were harvested and stained with fluorescein isothiocyanate (FITC)-labeled annexin V and propidium iodide (PI) using Annexin V-FITC Apoptosis Detection Kit (Beckman Coulter, CA, USA) following the manufacturer’s instructions. Apoptosis was measured using a FACSCalibur flow cytometer (BD Biosciences, CA, USA) and data were analyzed with CellQuest software (BD Biosciences, Franklin Lakes, NJ, USA).

### Measurements of malondialdehyde (MDA), glutathione (GSH), and superoxide dismutase (SOD)

Following transient transfection for 72 hour, SH-SY5Y cells were treated by OGD for 6 h, and cell lysates were collected to measure oxidative stress parameters using commercially available kits according to the manufacturer’s instructions. OxiSelect™ TBRS Assay Kit (MDA Quantitation) (STA-330), OxiSelect™ Superoxide Dismutase Activity Assay (STA-340), and OxiSelect™ Total Glutathione (GSSG/GSH) Assay Kit (STA-312) were purchased from Cell Biolabs, Inc. (USA) and used to determine the content of MDA, GSH, and the activity of SOD, respectively.

### Statistical analysis

All *in vitro* experiments were performed three times independently. Statistical tests were conducted using GraphPad Prism software. All data were tested by normal distribution analysis and statistical significance between multiple experimental groups was analyzed by one-way ANOVA and Tukey’s post hoc tests. The significance level was set at *P* < 0.05. Quantitative data were expressed as mean ± SD.

## Results

### Lumbosacral nerve root avulsion increased the level of Hsp27 protein in neurons of the anterior horn of the injured spinal cord

**Figure 1:**
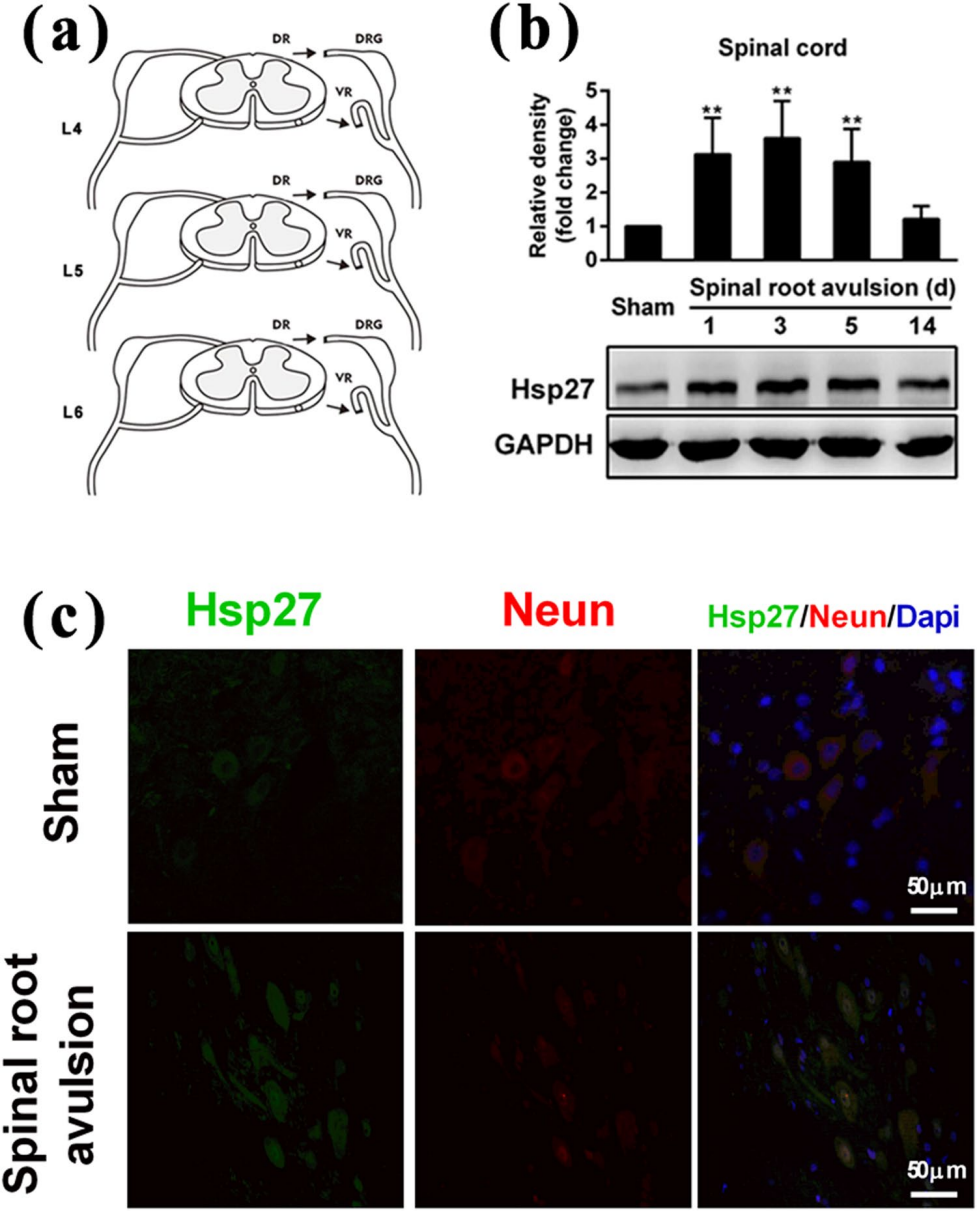
Effects of lumbosacral nerve root avulsion on the expression of Hsp27 protein in neurons of the anterior horn of the spinal cord. (**a**) Images of the procedure of lumbosacral nerve root avulsion injury in rats. Arrowheads indicate the injury site in the spinal cord (LNRA, lumbosacral nerve root avulsion; DR, dorsal root; DRG, dorsal root ganglion; VR, ventral root). (**b**) After the LNRA models of rats were established for 1, 3, 5, and 14 days, the Hsp27 protein level was detected by western blotting analysis in L4–L6 spinal cord tissue of the sham group (N = 4) and LNRA groups (N = 4). GAPDH was used as a loading control for western blotting. Hsp27 protein expression was quantified by densitometric analysis. (**c**) After LNRA injury for 3 days, Hsp27 (Green), NeuN (Red), and Dapi (Blue) were assessed by immunofluorescence staining in L4–L6 spinal cord tissue of the sham and LNRA groups. The representative image of neurons of the anterior horn of the spinal cord was shown. Scale bar, 50 μm. The results were expressed as fold change compared to the sham group. Each bar represents the mean ± SD. ^**^P < 0.01 versus the sham group.

First, we detected the level of Hsp27 protein in the injured spinal cord of rats after lumbosacral nerve root avulsion. The surgical procedure is shown in Fig. 1a. The tissue lysate of the injured spinal cord was prepared for western blotting analysis. As shown in Fig. 1b, the level of Hsp27 protein was significantly elevated in the injured spinal cord after nerve root avulsion surgery, peaked at 3 d (3.6-fold higher than the control, *p* < 0.01), and then decreased from 5 d to 14 d compared with the sham-operation group. Furthermore, the immunofluorescent staining assay showed that the level of Hsp27 protein was also elevated in neurons located in the anterior horn of the avulsion side of the spinal cord, at 3 d after surgery compared with the sham-operation group (Fig. 1c). These results indicated that the expression of Hsp27 protein was elevated in neurons of the anterior horn area of the injured spinal cord after lumbosacral nerve root avulsion.

### Oxygen-glucose deprivation increased the level of Hsp27 protein in SH-SY5Y neuroblastoma cells

**Figure 2:**
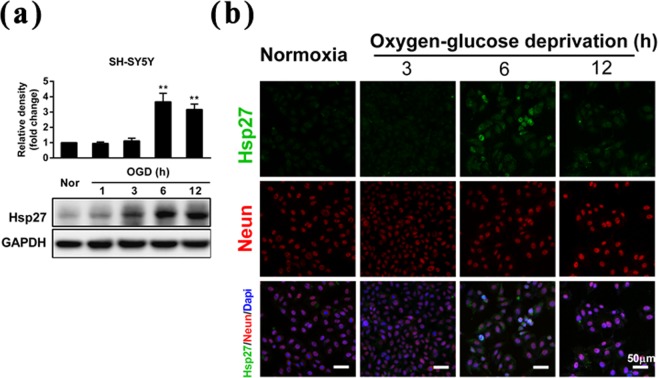
Effects of oxygen-glucose deprivation on the expression of Hsp27 protein in SH-SY5Y neuroblastoma cells. (**a**) Neuroblastoma cells were treated by oxygen-glucose deprivation (OGD) at the indicated time points, and then Hsp27 protein was detected by western blotting in SH-SY5Y cells. GAPDH was used as a loading control for western blotting. Hsp27 protein expression was quantified by densitometric analysis. (**b**) After neuroblastoma cells were treated by OGD for the indicated time points, Hsp27 (Green), NeuN (Red), and Dapi (Blue) were assessed by immunofluorescence staining in SH-SY5Y cells. A representative image of Hsp27 and NeuN staining is shown. Scale bar, 50 μm. The results were expressed as fold change compared to the normoxic (Nor) group. Each bar represents the mean ± SD. ^**^P < 0.01 versus the normoxic group.

The lumbosacral nerve root avulsion surgery resulted in unfavorable microenvironment for the neurons, including hypoxia, nutrition insufficiency, and hemorrhage. OGD was chosen to mimic the pathophysiological microenvironment generated by nerve root avulsion and we further determined the level of Hsp27 protein in SH-SY5Y cells under OGD conditions *in vitro*. The results of western blotting showed that the Hsp27 protein level increased significantly in SH-SY5Y cells treated with OGD for 6 h and 12 h (3.7 and 3.2-fold of the control, *p* < 0.01) (Fig. 2a). The immunofluorescent staining assay further confirmed that the expression of Hsp27 protein significantly increased in SH-SY5Y cells at 6 h after OGD (Fig. 2b). These results indicated that the expression of Hsp27 protein was elevated under OGD conditions in neuroblastoma cells.

### Up-regulation of Hsp27 protected neurons against apoptosis after lumbosacral nerve root avulsion

**Figure 3:**
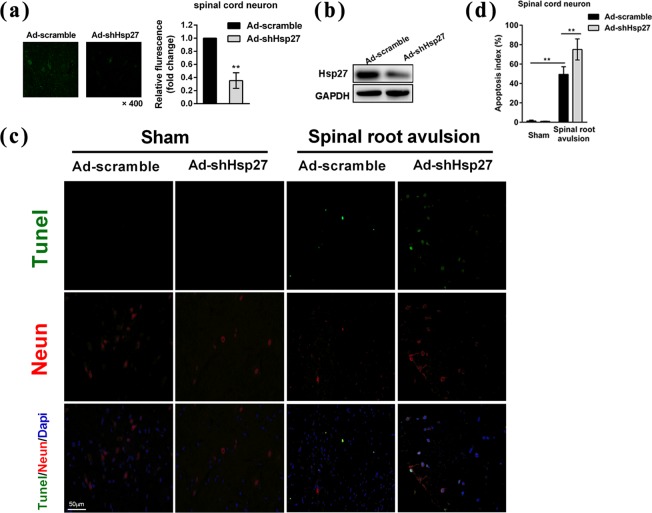
Silencing Hsp27 protein promoted lumbosacral nerve root avulsion-induced apoptosis in neurons of the anterior horn of the spinal cord. Transfection of the recombinant adenovirus carrying scramble RNA (Ad-scramble) or short hairpin RNA-Hsp27 (Ad-shHsp27) was performed by direct local injection into the L4-L6 spinal cord of LNRA rats or sham rats. Then, the surgery of lumbosacral nerve root avulsion in rats was performed after injection. The right L4-L6 spinal cord was resected for further assay 3 days after surgery. (**a**) Immunofluorescence staining and (**b**) western blotting were performed to confirm the effect of silencing the Hsp27 protein in the sham group. Fluorescence density was quantified using NIH ImageJ software. The results were expressed as fold change compared to the Ad-scramble group. (**c**) Tunel (Green), NeuN (Red), and Dapi (Blue) were assessed by immunofluorescence staining in the spinal cord of the sham and LNRA groups. (**d**) The apoptosis index was calculated by dividing the number of apoptotic neurons by the total number of neurons (red) located in the anterior horn of the spinal cord (N = 4 in each group). Each bar represents the mean ± SD. ^**^P < 0.01 versus the sham group transfected with Ad-scramble or the LNRA group transfected with Ad-scramble.

To assess the role of Hsp27 up-regulation in neurons of the anterior horn of the spinal cord after lumbosacral nerve root avulsion, we silenced Hsp27 protein in neurons by transfection of Ad-shHsp27 using direct local injection into the L4–L6 spinal cord. Immunofluorescent staining assay and western blotting confirmed the silencing effect of Hsp27 protein in neurons of the anterior horn of the spinal cord in the sham group (Fig. 3a,b). Furthermore, TUNEL staining was performed to detect apoptosis. We found that lumbosacral nerve root avulsion increased the number of apoptotic neurons of the anterior horn of the spinal cord in the scramble shRNA-transfected group and the apoptosis index increased to 49% after lumbosacral nerve root avulsion (*p* < 0.01) (Fig. 3b,c). Silencing of Hsp27 significantly increased the number of apoptotic neurons induced by lumbosacral nerve root avulsion compared with the scramble-transfected group and the apoptosis index increased to 74.9% (*p* < 0.01), indicating that knockdown of Hsp27 promoted lumbosacral nerve root avulsion-induced apoptosis (Fig. 3b,c). Together our data indicated that up-regulation of Hsp27 protected neurons against lumbosacral nerve root avulsion-induced apoptosis *in vivo*.

### Up-regulation of Hsp27 protected SH-SY5Y neuroblastoma cells against apoptosis after oxygen-glucose deprivation

**Figure 4:**
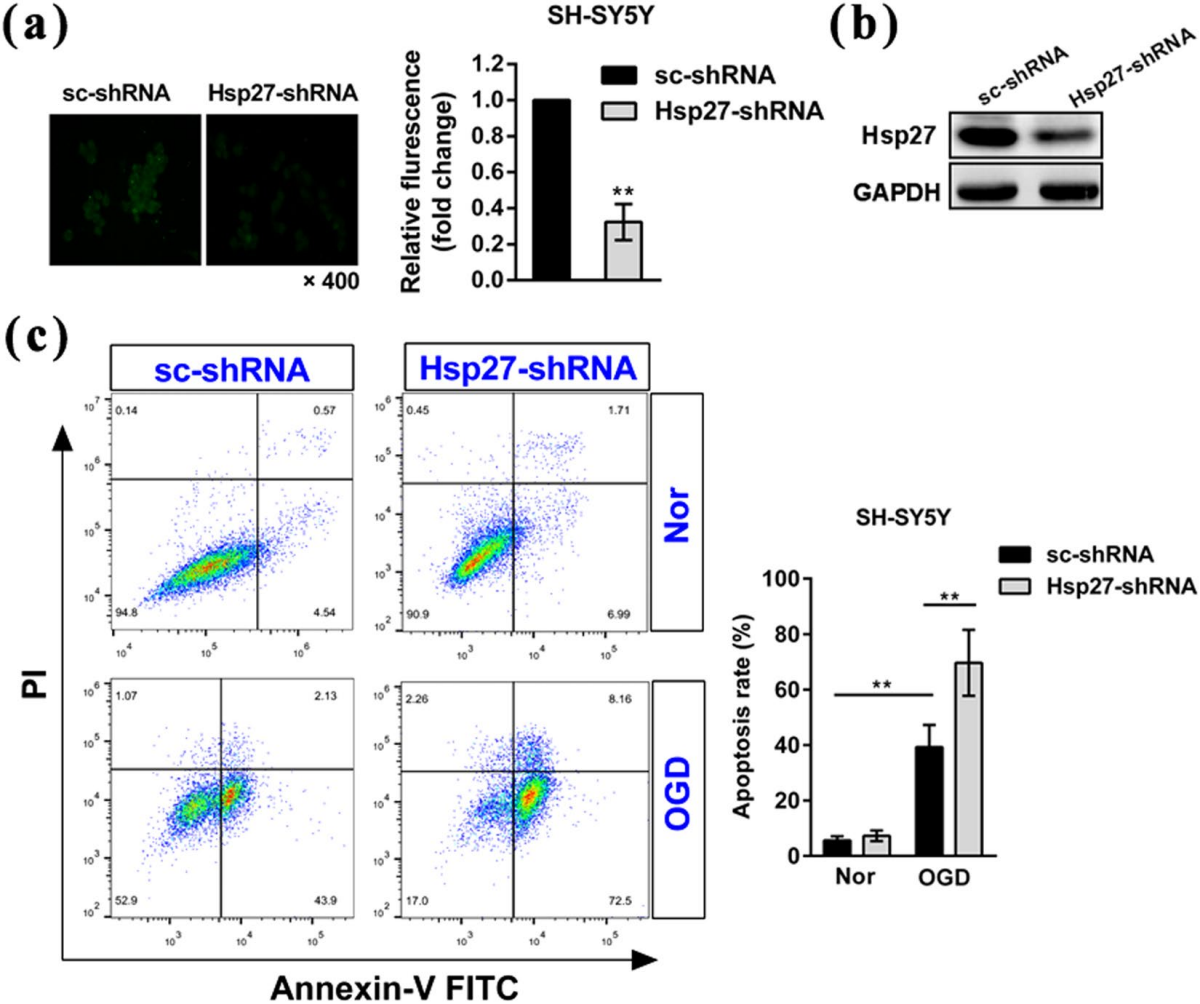
Suppression of Hsp27 protein promoted the oxygen-glucose deprivation-induced apoptosis in SH-SY5Y neuroblastoma cells. Neuroblastoma cells were transfected with the indicated scramble RNA (sc-shRNA) or short hairpin RNA-Hsp27 (Hsp27-shRNA) for 72 h, and then cells were cultured in normoxic conditions or treated with OGD for 6 h. (**a**) Immunofluorescence staining and (**b**) western blotting was performed to confirm the effect of suppression of Hsp27 protein in normoxic cells. Fluorescence density was quantified using NIH ImageJ software. The results were expressed as fold change compared to the cells transfected with sc-shRNA. (**c**) Apoptosis was determined by Annexin-V FITC and PI staining and flow cytometry. Annexin-V (+)/PI (−) cells are early apoptotic cells and Annexin-V (+)/PI (+) cells are late apoptotic cells. The FACS analysis graphs (left) are representative of three independent experiments. The rate of apoptosis was early apoptosis percentage plus late apoptosis percentage. Data were expressed as mean ± SD. ^**^P < 0.01 versus the scramble shRNA-transfected cells under normoxia (Nor) or the scramble shRNA-transfected cells treated by OGD.

We further determined the role of OGD-induced up-regulation of Hsp27 by silencing the expression of Hsp27 protein *in vitro*. As shown in Fig. 4a,b, the silencing effect of Hsp27 protein in SH-SY5Y cells was confirmed by the immunofluorescent staining assay and western blotting. Furthermore, annexin-V FITC/PI staining and flow cytometry were performed to detect apoptosis. We found that OGD treatment significantly increased the apoptosis rate in scramble shRNA-transfected SH-SY5Y cells (Fig. 4c). Suppression of Hsp27 protein significantly increased OGD-induced apoptosis rate in transfected SH-SY5Y cells, indicating that knockdown of Hsp27 promoted OGD-induced apoptosis (Fig. 4c). These results indicated that up-regulation of Hsp27 also protected neuroblastoma cells against OGD-induced apoptosis *in vitro*.

### Hsp27 regulated the oxygen-glucose deprivation-induced apoptosis in primary rat neurons

**Figure 5:**
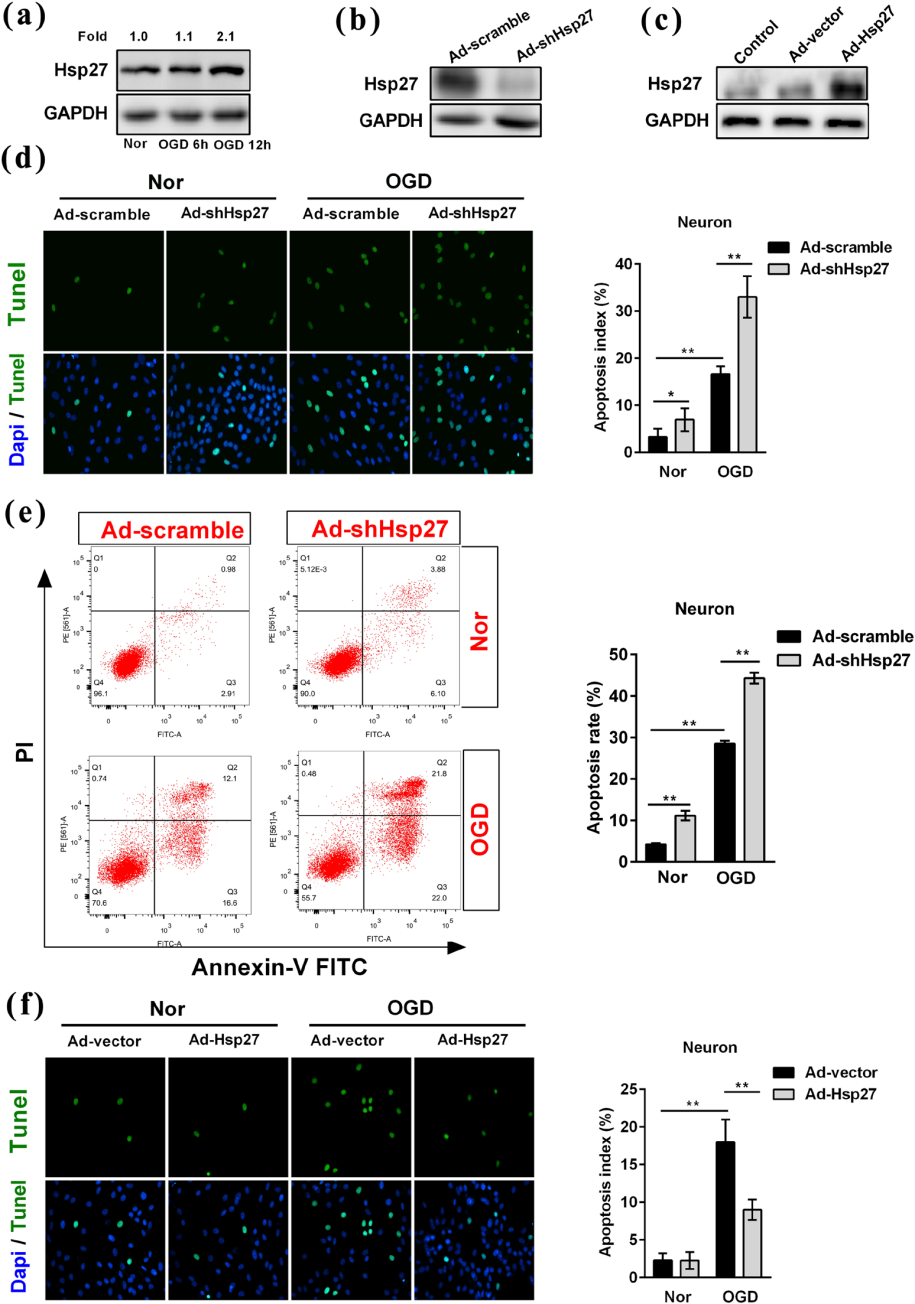
Hsp27 regulated the oxygen-glucose deprivation-induced apoptosis in primary rat neurons. (**a**) Primary neurons were treated by oxygen-glucose deprivation (OGD) at the indicated time points, and then Hsp27 protein was detected by western blotting in primary neurons. Primary neurons were transfected with the recombinant adenovirus carrying scramble RNA (Ad-scramble), short hairpin RNA-Hsp27 (Ad-shHsp27), empty vector (Ad-vector) or Hsp27 expression plasmid (Ad-Hsp27) for 48 h, and then neurons were cultured under normoxic conditions (Nor) or treated with OGD for another 12 h. Western blotting was performed to confirm the knock-down (**b**) and over-expression (**c**) effects of Hsp27 protein in normoxic neurons. (**d**,**f**) Tunel (Green) and Dapi (Blue) were assessed by immunofluorescence staining in transfected neurons under normoxic and OGD condition. The apoptosis index was calculated by dividing the number of apoptotic neurons (Green) by the total number of neurons (Blue). (**e**) Apoptosis was determined by Annexin-V FITC and PI staining and flow cytometry. Annexin-V (+)/PI (−) cells are early apoptotic cells and Annexin-V (+)/PI (+) cells are late apoptotic cells. The FACS analysis graphs are representative of three independent experiments. The rate of apoptosis was early apoptosis percentage plus late apoptosis percentage. Data were expressed as mean ± SD. ^*^P < 0.05 versus the scramble- or vector-transfected cells under normoxia, ^**^P < 0.01 versus the scramble- or vector-transfected cells under normoxia or the scramble- or vector-transfected cells treated by OGD.

Next, we explored the role of Hsp27 in oxygen-glucose deprivation-induced apoptosis in primary neurons. We also found that OGD treatment induced a 2.1-fold increase in the protein level of Hsp27 at 12 h in primary rat neurons (Fig. 5a). As shown in Fig. 5b,c, the knock-down and over-expression effects of Hsp27 protein in normoxic neurons were confirmed by western blotting analysis. We found that OGD treatment significantly increased the apoptosis rate in primary rat neurons transfected with Ad-scramble (Fig. 5d,e). Suppression of Hsp27 protein significantly increased apoptosis rate in normoxic neurons and OGD-treated neurons, indicating that knockdown of Hsp27 promoted neuron apoptosis (Fig. 5d,e). However, over-expression of Hsp27 significantly reduced OGD-induced apoptosis rate (Fig. 5f). These results indicated that Hsp27 protected primary rat neurons against OGD-induced apoptosis *in vitro*.

### Up-regulation of Hsp27 protected SH-SY5Y cells against OGD-induced apoptosis by suppressing oxidative stress reactions

**Figure 6:**
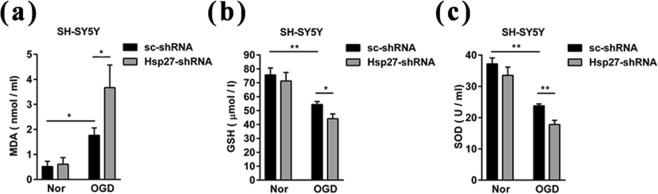
Suppression of Hsp27 protein promoted the oxidative stress reaction in SH-SY5Y neuroblastoma cells. Neuroblastoma cells were transfected with the indicated scramble RNA (sc-shRNA) or short hairpin RNA-Hsp27 (Hsp27-shRNA) for 72 h, and then cells were cultured in normoxia or treated with OGD for 6 h. Cell lysates were prepared for MDA (**a**), GSH (**b**), and SOD (**c**) detection as described as in “Materials and Methods”. Data were expressed as mean ± SD of three independent experiments. ^*^P < 0.05, ^**^P < 0.01 versus the sc-shRNA -transfected cells in normoxia or the sc-shRNA-transfected cells treated by OGD.

Oxidative stress has been implicated in the process of apoptosis^18^, so we next determined the content of MDA, a product of lipid peroxidation that usually acts an indicator for oxidative stress, and measured two anti-oxidant enzymes, GSH content and SOD activity, to assess the oxidative stress reactions induced by OGD in SH-SY5Y cells. We found that OGD treatment significantly increased the content of MDA in scramble shRNA-transfected cells (Fig. 6a). Knockdown of Hsp27 increased the effect of OGD-induced augmentation of MDA (Fig. 6a). As shown in Fig. 6b,c, OGD treatment reduced the content of GSH and the activity of SOD in scramble shRNA-transfected cells, and knockdown of Hsp27 enhanced the effect of OGD-reduced GSH content and SOD activity. These results indicated that up-regulation of Hsp27 inhibited OGD-induced apoptosis by suppressing oxidative stress reactions.

### Up-regulation of Hsp27 protein contributed to the activation of NF-κB signaling in primary rat neurons under OGD condition

**Figure 7:**
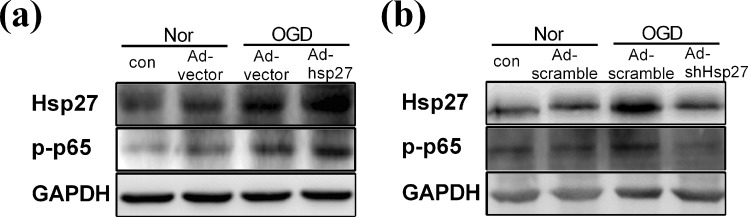
Up-regulation of Hsp27 protein contributed to the activation of NF-κB signaling in primary rat neurons under OGD condition. (**a**,**b**) Primary neurons were transfected with the indicated recombinant adenovirus carrying empty vector (Ad-vector), Hsp27 expression plasmid (Ad-Hsp27), scramble RNA (Ad-scramble) or short hairpin RNA-Hsp27 (Ad-shHsp27) for 48 h, and then neurons were cultured in normoxic conditions (Nor) or treated with OGD for another 12 h. Total cell lysates were harvested, and then Hsp27 and p-p65 protein were detected by western blotting. GAPDH was used as a loading control for western blotting.

We further investigated the mechanism underlying the anti-apoptotic role of Hsp27 in OGD-induced primary neurons. As shown in Fig. 7a, OGD treatment up-regulated the Hsp27 expression and increased the phosphorylation of p65, meanwhile over-expression of Hsp27 markedly enhanced the phosphorylated level of p65 under OGD condition, indicating that up-regulation of Hsp27 activated NF-κB signaling in primary rat neurons under OGD condition. However, suppression of Hsp27 markedly decreased the phosphorylation of p65 and abrogated the OGD-induced NF-κB activation (Fig. 7b). These results indicated that up-regulation of Hsp27 might inhibit OGD-induced apoptosis through activating NF-κB signaling pathway.

## Discussion

Spinal nerve root avulsion has been proven to cause degeneration of adult motor neurons^19^. As the survival of motor neurons is essential for functional recovery after nerve root avulsion, progressive motor neuron death exerts a significant influence on the prognosis. Neurons in the spinal cord have been shown to undergo apoptosis after lumbosacral nerve root avulsion^9^, which leads to a drastic decrease in the survival rate of the motor neurons. Previous studies showed that spinal cord injury, another serious central nervous system injury, resulted in the activation of the inflammatory response and apoptosis^20,21^. Meanwhile, Hsp27, which was induced by stress and protected against cellular injuries in various cell types including neurons^22^, was also upregulated after spinal cord transection injury^23^, autoimmune encephalomyelitis^24^, and heat acclimation^25^ in rats. However, the expression of Hsp27 and its role in spinal nerve root avulsion remains ambiguous.

In the present study, we have shown that spinal nerve root avulsion led to an up-regulation of Hsp27 expression in the anterior horn area of the injured spinal cord. Hsp27 expression was elevated on the avulsion side of the spinal cord at 1 and 3 d after surgery, peaked at 3 d, and then began decreasing from 5 d to 14 d compared with the sham-operation group. We hypothesized that lumbosacral nerve root avulsion caused damage to the spinal cord, resulting in an adverse microenvironment for the neurons. Consequently, OGD was chosen to mimic the pathophysiological microenvironment *in vitro*. Hsp27 expression increased in SH-SY5Y cells and primary rat neurons at 6 h and 12 h after OGD. These results indicated that Hsp27 expression was elevated in the adverse microenvironment. The upregulation of Hsp27 plays cytoprotective roles under different conditions such as cerebral ischemia^26,27^, myocardial infarction^28^, cardiac dysfunction^29^, neonatal nerve crush^30^, and amyotrophic lateral sclerosis^31^. The cytoprotective effects of Hsp27 might be associated with its role as a molecular chaperone, regulation of the cytoskeleton, modulation of intracellular redox potential, and especially anti-apoptotic activity^32^. In the present study, to assess the role of Hsp27 up-regulation in neurons after lumbosacral nerve root avulsion, we silenced Hsp27 expression in neurons using Ad-shHsp27 by direct local injection into the L4-L6 spinal cord. Our findings suggested that up-regulation of Hsp27 protected neurons against SRA-induced apoptosis *in vivo*. We further determined the role of OGD-induced up-regulation of Hsp27 by silencing the expression of Hsp27 protein *in vitro*, and the results showed that suppression of Hsp27 protein significantly increased the OGD-induced apoptosis rate in transfected SH-SY5Y cells and primary rat neurons. However, up-regulation of Hsp27 significantly decreased OGD-induced apoptosis rate. These data suggest that HSP27 plays an anti-apoptotic role after lumbosacral nerve root avulsion.

We further explored the potential mechanisms for the anti-apoptotic role of Hsp27 after lumbosacral nerve root avulsion. Recent evidence has shown that Hsp27 could inhibit apoptosis through the interaction of Hsp27 with protein kinase B (Akt). Hsp27-mediated activation of Akt contributes to the resistance to apoptosis in cells expressing high levels of Hsp27^33^. In addition, Hsp27 was proven to inhibit apoptosis through direct inhibition of caspase activation. Garrido *et al*.^34^ found that HSP27 inhibited etoposide-induced apoptosis by preventing activity of caspase-9 in human leukemic cells. Furthermore, Concannon *et al*.^35^ showed that Hsp27 inhibited caspase-3 activity to prevent the function of the apoptosome complex in human Jurkat cells. Evidence has accumulated that Hsp27 can protect L929 cells against ROS generated by either TNF-α or oxidative stress^36^. In this study, we assessed the oxidative stress reactions and activation of NF-κB signaling induced by OGD in SH-SY5Y cells and primary rat neurons respectively. The results indicated that up-regulation of Hsp27 inhibits OGD-induced apoptosis by suppressing oxidative stress reactions in SH-SY5Y cells and activating NF-κB signaling pathway in primary rat neurons.

In conclusion, we have demonstrated that the expression of Hsp27 was upregulated after lumbosacral nerve root avulsion and oxygen-glucose deprivation. Hsp27 plays an anti-apoptotic role under these conditions by suppressing oxidative stress reactions. These findings indicated that Hsp27 plays a key role in resistance to lumbosacral nerve root avulsion-induced neuron apoptosis and may prove to be a strategy for improving prognosis after lumbosacral nerve root avulsion.
